# Higher Protein Intake Does Not Improve Lean Mass Gain When Compared with RDA Recommendation in Postmenopausal Women Following Resistance Exercise Protocol: A Randomized Clinical Trial

**DOI:** 10.3390/nu9091007

**Published:** 2017-09-12

**Authors:** Luana T. Rossato, Paula C. Nahas, Flávia M. S. de Branco, Fernanda M. Martins, Aletéia P. Souza, Marcelo A. S. Carneiro, Fábio L. Orsatti, Erick P. de Oliveira

**Affiliations:** 1 School of Medicine, Federal University of Uberlandia (UFU), Av. Pará, n° 1720, Bloco 2U, Campus Umuarama, Uberlandia 38400-902, Minas Gerais, Brazil; luanathrossato@hotmail.com (L.T.R.); nahaspaula6@gmail.com (P.C.N.); fla-msb@hotmail.com (F.M.S.d.B.); 2 Exercise Biology Research Group (BioEx), Federal University of Triangulo Mineiro (UFTM), Uberaba 38061-500, Minas Gerais, Brazil; fernanda_mmartins@hotmail.com (F.M.M.); teia_depaula_souza@hotmail.com (A.P.S.); marcelo__mrcl@hotmail.com (M.A.S.C.); fabiorsatti@gmail.com (F.L.O.); 3 Department of Sport Sciences, Federal University of Triangulo Mineiro (UFTM), Uberaba 38061-500, Minas Gerais, Brazil

**Keywords:** body composition, protein, RDA recommendations, leucine intake, postmenopausal women

## Abstract

The aim of this study was to evaluate the effect of a higher protein intake on lean body mass (LBM) gain in postmenopausal women practicing resistance exercise and compare it to the Recommended Dietary Allowance (RDA) recommendation. Twenty-three postmenopausal women (63.2 ± 7.8 years) were randomized into two groups. The group with higher protein intake (*n* = 11) (HP) received a dietary plan with ~1.2 g·kg^−1^·day^−1^ of protein, while the normal protein (NP) group (*n* = 12) was instructed to ingest ~0.8 g·kg^−1^·day^−1^ of protein (RDA recommendation). Both groups performed the same resistance training protocol, 3 times a week, with progression of the number of sets (from 1 to 6 sets) and 8–12 repetitions. The intervention occurred over 10 weeks. Body composition evaluation was performed by dual-energy X-ray absorptiometry. The diet was evaluated by nine 24-h food recall summaries over the course of the study. During the intervention period, the HP group presented a higher protein (1.18 ± 0.3 vs. 0.87 ± 0.2 g·kg^−1^·day^−1^, *p* = 0.008) and leucine (6.0 ± 1.4 vs. 4.3 ± 0.9 g/day, *p* < 0.001) intake than the NP group, respectively. At the end of the intervention, there were increases in LBM both in HP (37.1 ± 6.2 to 38.4 ± 6.5 kg, *p* = 0.004) and in NP (37.6 ± 6.2 to 38.8 ± 6.4 kg, *p* < 0.001), with no differences between the groups (*p* = 0.572). In conclusion, increased protein intake did not promote higher LBM gain when compared to RDA recommendation in postmenopausal women performing resistance exercise during 10 weeks. This trial was registered at ClinicalTrials.gov as NCT03024125.

## 1. Introduction

Aging is associated with a gradual and progressive loss of muscle mass and function [1,2]. In addition, muscle protein synthesis is attenuated in older individuals in the postprandial period, characterizing a process called anabolic resistance [3,4]. Middle-aged adults have similar negative metabolic consequences of aging [5], and postmenopausal women are included in this context. This population deserves special attention because a decrease in serum estrogen levels is observed during this period [6,7], which seems to have direct and indirect effects on skeletal muscle [8], such as inhibiting the production of catabolic cytokines, including interleukins-1 and -6 and tumor necrosis factor-α [9]. These effects may lead to greater muscle and strength loss, concomitantly with greater body fat accumulation [10]. Resistance training and adequate protein intake are known strategies to increase lean body mass (LBM) [11,12]. A greater intake of high-quality proteins seems to promote additional muscle gain in resistance exercise protocol [13]. Thus, the association of both interventions seems to be an effective strategy to promote LBM gain [14].

In this context, the protein intake proposed by the Recommended Dietary Allowance (RDA) for older adults, including postmenopausal women, is 0.8 g·kg^−1^·day^−1^ [15]. However, current studies have shown that a higher amount of protein, ~1.2 g·kg^−1^·day^−1^, seems to be more effective in stimulating muscle protein synthesis (MPS) or muscle maintenance [16,17,18,19,20,21]. This implies that the RDA recommendation seems to be insufficient for older individuals, although this is not a consensus [22]. A recent study compared the intake of 1.2 g·kg^−1^·day^−1^ to 0.8 g·kg^−1^·day^−1^ in older men, over the course of 3 days, and no difference in acute MPS stimulation after exercise was observed between groups [22]. However, considering that acute measurements of MPS do not seem to be directly associated with longer-term changes in muscular hypertrophy [23], long-term studies are needed to evaluate the effect of this protein intake on LBM gain to confirm these results and include postmenopausal women in the target group.

Therefore, the aim of our study was to compare the effect of higher protein intake (~1.2 g·kg^−1^·day^−1^) with RDA recommendations (~0.8 g·kg^−1^·day^−1^) on LBM gain in postmenopausal women following resistance exercise protocol. We hypothesized that higher protein intake would enhance LBM gain associated with resistance exercise.

## 2. Methods

### 2.1. Research Participants

This trial was a single-blind, randomized, parallel prospective clinical trial, conducted at the Federal University of Uberlandia and the Federal University of Triangulo Mineiro, Minas Gerais, Brazil. Inclusion criteria for participants were postmenopausal women (cessation of menstruation for at least 1 year; self-reported) and a sedentary lifestyle. Those who presented orthopedic limitation, any stage of kidney disease, alcoholic habits, and/or were receiving hormone replacement were excluded. The study was approved by Federal University of Uberlandia Research Ethics (protocol number 1.733.512) and Federal University of Triangulo Mineiro Research Ethics Committees (protocol number 1.090.676). This research was registered at ClinicalTrials.gov as NCT03024125.

**Figure 1:**
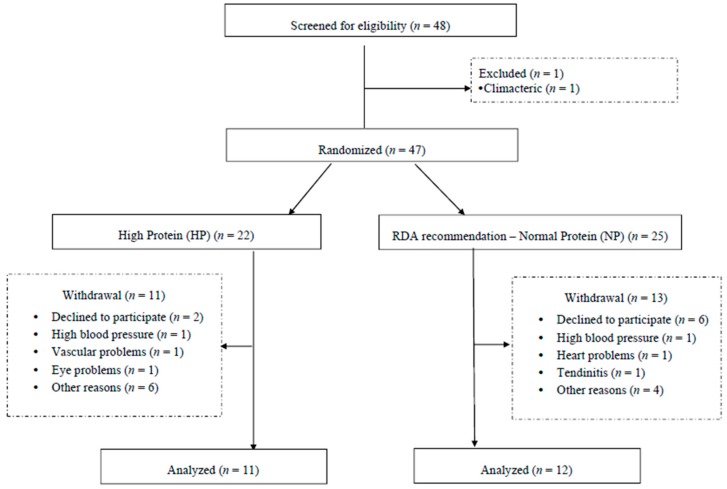
Randomization of the individuals in the study.

Initially, 48 participants were recruited and 1 volunteer was excluded from the study. Once consent was obtained, all included participants were randomly assigned by MedCalc^®^ software (version 11.1, MedCalc Software, Mariakerk, Belgium) into 2 groups: RDA protein recommendations (~0.8 g·kg^−1^·day^−1^) (normal protein, (NP)) and high protein (HP) (~1.2 g·kg^−1^·day^−1^). Due to losses that occurred during the intervention, the HP group contained 11 individuals and the NP group contained 12 individuals (Figure 1). Baseline values of LBM were the variable chosen for randomization and the analysis was performed by an independent researcher.

An a priori power analysis was performed (G*Power v. 3.0.10, Heinrich-Heine-University Düsseldorf, Düsseldorf, Germany [24]) for an *F* test (repeated measures, within-between interaction factors for 2 time points) to calculate the required number of participants in each group. On the basis of a statistical power (1–*β* err prob) of 0.80, a moderately large effect size (0.5), and an overall level of significance of 0.05, 12 subjects were required for this study.

### 2.2. Study Design

**Figure 2:**
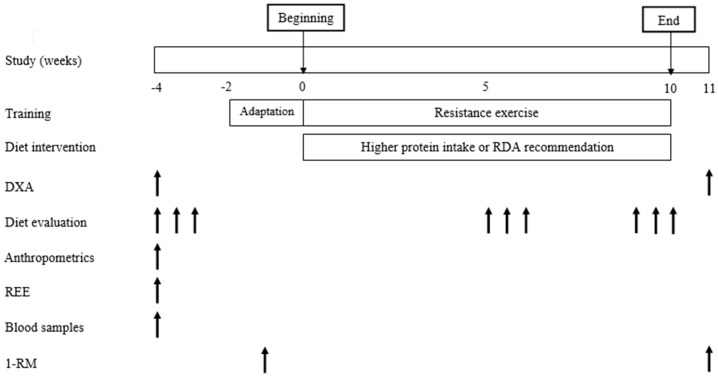
Schematic overview diagram of the study protocol.

Before the study commenced, body composition (dual-energy X-ray absorptiometry (DXA)), dietary habits, anthropometric parameters, strength, resting energy expenditure (REE) and blood samples were assessed. The evaluation of these analyses was performed 2 weeks before the intervention. Additionally, a 2-week familiarization period was done to ensure that the postmenopausal women were able to perform all resistance exercises safely and correctly. Week 1 consisted of the familiarization period and week 2 consisted of 1 repetition maximum test (1-RM). Resistance training and diet intervention were performed during 10 weeks. Dietary intake was also assessed at 5–6 weeks and 9–10 weeks. At the end of the study, anthropometric measurements, body composition, and strength were re-evaluated. The study protocol is described in Figure 2.

### 2.3. Assessments

#### 2.3.1. Anthropometry

Body mass and height were measured according to the protocol proposed by Lohman et al. [25] (balance and stadiometer Líder^®^, Araçatuba, Brazil), followed by body mass index (BMI) calculation.

#### 2.3.2. Body Composition

Total lean mass, leg lean mass, total fat mass, and trunk fat mass were assessed using DXA (Lunar iDXA^®^, GE Healthcare, Madison, WI, USA) and quantified by software Encore version 14.10 (GE Medical Systems, Madison, WI, USA). Twenty-four hours before the evaluation, volunteers were instructed to ingest 2 L of water to standardize the level of muscle hydration and were oriented to 8–10 h of fasting, as previously reported [26]. The volunteers wore light and comfortable clothes without the presence of metal objects. The equipment was used manually and all analyses were performed by the same researcher. The body scan was divided into arms, legs, trunk and head.

### 2.4. Strength Measurement

Muscle strength was analyzed by 1-RM. Before the 1-RM test, all women participated in 3 sessions, on non-consecutive days, to learn the exercise techniques. The 1-RM test was performed the following week. Initially, a warm-up was performed using a subjective load, determined during the familiarization, with 8–10 repetitions of 40–60% of 1-RM. After 1 min of rest, the load was increased to 60–80% of 1-RM and, 3–5 repetitions were performed. After 3–5 min of rest, the load was considerably increased, and the participants were encouraged to overcome resistance using full motion. If the subjects performed only 1 complete movement (full range of motion), the 1-RM was established and the loads used in these exercises were adopted as the maximum strength. When the women were unable to perform the movement or performed more than 1 repetition, a new attempt was performed with a lighter or heavier load, respectively. One repetition maximum was determined with a maximum of 5 attempts and, the women rested 3–5 min between attempts. The 1-RM test was performed for bench press and leg extension and a trained examiner performed the 1-RM measurements.

### 2.5. Resting Energy Expenditure (REE) and Total Energy Expenditure (TEE)

Resting energy expenditure was measured by indirect calorimetry (open circuit mixing-chamber system), using the VO2000 metabolic measurement system (MedGraphics, Ann Arbor, MI, USA). The device was turned on 30 min before the examination for heating, proper stabilization and calibration of the O_2_ and CO_2_ analyzers with the ambient air [27]. The volunteers started the test after 12 h of overnight fast, 6–8 h of sleep, without intense physical activity in the previous 48 h of the examination and 24 h without caffeine consumption before the test. The test was performed in a quiet, temperature-controlled room [28]. There was a 10-min acclimatization period for reading stabilization, and then VO_2_ and VCO_2_ were measured for a period of 20 min. Mean values of these variables were inserted into the Weir equation [29] for REE measurement. Four women (2 of each group) did not complete the test and REE was estimated by the FAO/WHO/UNU equation [30].

For the calculation of total energy expenditure, REE was measured or calculated (as described above) and the value of 1.3 was used for all calculations as the factor activity (FA) (concerning daily activities). For calculation of metabolic equivalent (MET), we used the value 5.5 MET for resistance exercise [31]. After estimating an effective training time, MET training was calculated and then multiplied by 3 (number of times that physical training occurred a week) and the resulting value was divided by 7 (corresponding to the days of the week). The following formula was used: TEE = (REE × FA) + MET [32].

### 2.6. Dietary Assessment

Dietary intake was assessed by 24-h food recall. Three recalls were performed at baseline (weeks −3 and −4) and at 5–6 weeks and 9–10 weeks of intervention, with a total of 9 dietary recalls. Arithmetic mean was held from 6 food recalls performed during the intervention for better representation of the dietary habits of the volunteers during the intervention period. The food recalls were performed on non-consecutive days, and referred to 2 weekdays and 1 weekend day at each time point in the study. Two food recalls were conducted in person and the others via phone call after adequate familiarization. Analyses of food data were performed using Dietpro^®^ software (version 5.7i, Agromídia Softwares^®^, Minas Gerais, Brazil) and the United States Department of Agriculture (USDA) [33,34] food composition table was used. In addition, nutrition labels of manufacturers were also utilized.

The distribution of protein and leucine throughout the day was evaluated according to the meals described by volunteers and divided into: breakfast, morning snack, lunch, afternoon snack, dinner and supper. No fixed meal times were set. The 24-h food recall was evaluated according to the pre-established meals, taking into account the time and type of food consumed. We also evaluated the protein intake before and after resistance training.

### 2.7. Blood Samples

Blood samples (16 mL) were collected between 7:30 a.m. and 9:00 a.m. after an overnight fast (8–10 h), and placed in a dry tube with gel separator or EDTA (vacuum-sealed system, Vacutainer^®^, BDH, Poole, Dorset, UK) Urea and creatinine were analyzed by automated colorimetrics. These variables were measured to evaluate renal function at baseline.

### 2.8. Experimental Protocol

#### 2.8.1. Dietary Intervention

After randomization of the participants into 2 groups, individualized dietary plans, with their lists of food substitutions were given to all participants. All diets prescribed were normocaloric and eucaloric. The NP group contained ~0.8 g protein·kg^−1^·day^−1^ in their dietary plans while the group HP contained ~1.2 g protein·kg^−1^·day^−1^. The amount of carbohydrate was the same in both groups (50% of total caloric value). Decreased lipids were offered in the HP group (15% of total calories) and increased lipids were offered in the NP group (25–30% of total calories), for calorie adjustment. The participants were blinded for NP and HP groups. In order to reach the proposed amount of protein in each group, high quality protein, such as meat, fish, eggs, milk and other dairy products were recommended for the HP group. In addition, both groups were instructed to ingest similar amounts of protein after training (20–30 g), since protein intake after the exercise seems to be an important timing for hypertrophy [35] and could be a confounding factor for evaluating the effect of higher protein intake in 24 h.

#### 2.8.2. Resistance Training

The resistance training was conducted in the Public Health and Physical Activity Centre at the Federal University of Triangulo Mineiro and the protocol targeted muscle hypertrophy and followed the recommendations of the American College of Sports Medicine [36]. The minimum acceptable frequency for training was 70% and resistance training was realized 3 times a week with, at least 48 h of rest between sessions. Initially, the women started with 10 min of warm-up (walking). Dynamic resistance exercises were realized for the upper and lower limbs, including guided squat (free weight), leg curl (machine), leg extension (machine), bench press (free weight), rowing (machine), pull down (machine), triceps pulley and arm curl (free weight). All volunteers attended a 1-week familiarization period with lighter loads in order to learn the techniques. The load of 12-RM was applied for all resistance exercise and 8–12 repetitions were completed in each set. The interval between sets and exercises was 60 s. The resistance training was carried out for 10 weeks [37]. The groups started with 1 series of each exercise in the first week and increased a set per week up to 6 series for all exercises. When they reached the 6 series (in the sixth week), they kept this volume until the 10th week. In the sixth week, there was a readjustment of the load for volunteers who were able to perform more than 12 RM. The load was increased until the volunteer remained in the range of 8–12 repetitions. During the training sessions, the subjects were instructed to perform the eccentric and concentric action in 1 s each. The exercises were supervised during all the protocol by trained professionals, who were blind to nutritional intervention. The resistance training was performed in the morning or in the afternoon and equally distributed for NP and HP groups.

#### 2.8.3. Statistics

The data distribution was determined using the Shapiro-Wilk test. The independent *t*-test or Mann-Whitney *U*-test was used to compare groups at baseline. Generalized Estimating Equation (GEE) with sequential Sidak post hoc was performed to compare groups and moments, and to evaluate the interaction between time × treatment. Delta (Δ), final minus initial value, was calculated for protein (g·kg^−1^·day^−1^) and leucine (g/day) intake, total lean mass (TLM) (kg), leg lean mass (LLM) (kg), total fat mass (TFM) (kg), trunk fat mass (TrFM) (kg), bench press RM and leg extension RM. The values were described in mean ± standard deviation for the *t*-test and GEE, and in median (interquartile range) for Mann-Whitney. In addition, we performed a linear regression analysis evaluating all individuals (*n* = 23) to associate the Δ protein intake (g) with Δ TLM and Δ LLM. A *p*-value of < 0.05 was adopted for statistical significance and SPSS software (version 20.0, IBM Corp, New York, NY, USA) was used for statistical analysis.

## 3. Results

### 3.1. Baseline Characteristics

No differences were found for age, anthropometric measurements, body composition, strength, biochemical parameters, and energy expenditure between groups at baseline (Table 1).

### 3.2. Dietary Intake

At baseline, both groups presented the same intake for all evaluated diet components, including protein. No changes were observed for calories, carbohydrate (g) and lipid (g and %) intake between groups or moments. Carbohydrate intake (%) remained the same in NP group, but decreased in the HP group during the intervention, however, no differences were found between groups. The HP group increased isoleucine and valine during intervention (Table 2).

**Figure 3:**
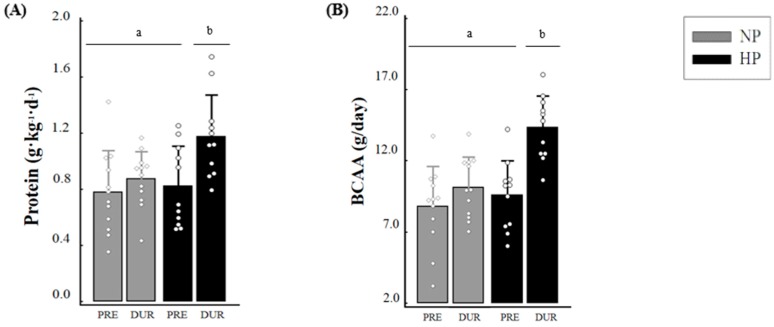

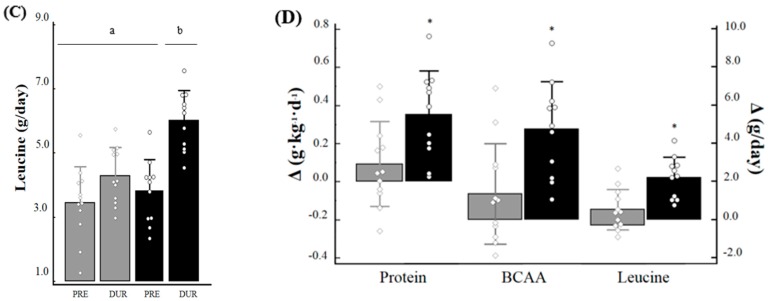
Daily intake of protein (**A**), BCAA (**B**) and leucine (**C**) at pre and during the study and respective deltas (**D**). Notes: DUR: during; Δ: delta values; BCAA: branched-chain amino acid. Generalized Estimating Equation (GEE) with sequential Sidak post hoc was performed to evaluate the intake between groups and moments; independent *t*-test was performed to evaluate the difference between deltas; *: different (*p* < 0.05) from delta NP. Different letters (a, b) mean *p* < 0.05. Circle sign is the value of each individual of the study.

The NP group maintained the intake of protein (0.78 ± 0.3 vs. 0.87 ± 0.29 g·kg^−1^·day^−1^, *p* = 0.332), branched-chain amino acid (BCAA) (8.8 ± 2.81 vs. 10.15 ± 2.14 g/day, *p* = 0.117) and leucine (3.46 ± 1.12 vs. 4.3 ± 0.88 g/day, *p* = 0.396), whereas the HP group increased the intake of these dietary components during the study (protein: 0.82 ± 0.29 vs. 1.18 ± 0.3 g·kg^−1^·day^−1^, *p* < 0.001; BCAA: 9.59 ± 2.42 vs. 14.34 ± 2.25 g/day, *p* < 0.001; leucine: 3.82 ± 0.99 vs. 6.02 ± 0.93 g/day, *p* < 0.001). The values of protein (*p* = 0.013), BCAA (*p* = 0.005) and leucine (*p* = 0.006) were higher for the HP group when compared to the NP group during the study. The values for Δ protein (0.35 ± 0.23 vs. 0.09 ± 0.22 g·kg^−1^·day^−1^, *p* = 0.013), Δ BCAA (4.76 ± 2.48 vs. 1.35 ± 2.64 g/day, *p* = 0.005), and Δ leucine (2.20 ± 1.07 vs. 0.83 ± 1.06 g/day, *p* = 0.006) were higher in the HP compared with the NP, respectively (Figure 3).

### 3.3. Distribution of Protein and Leucine during the Day

**Figure 4:**
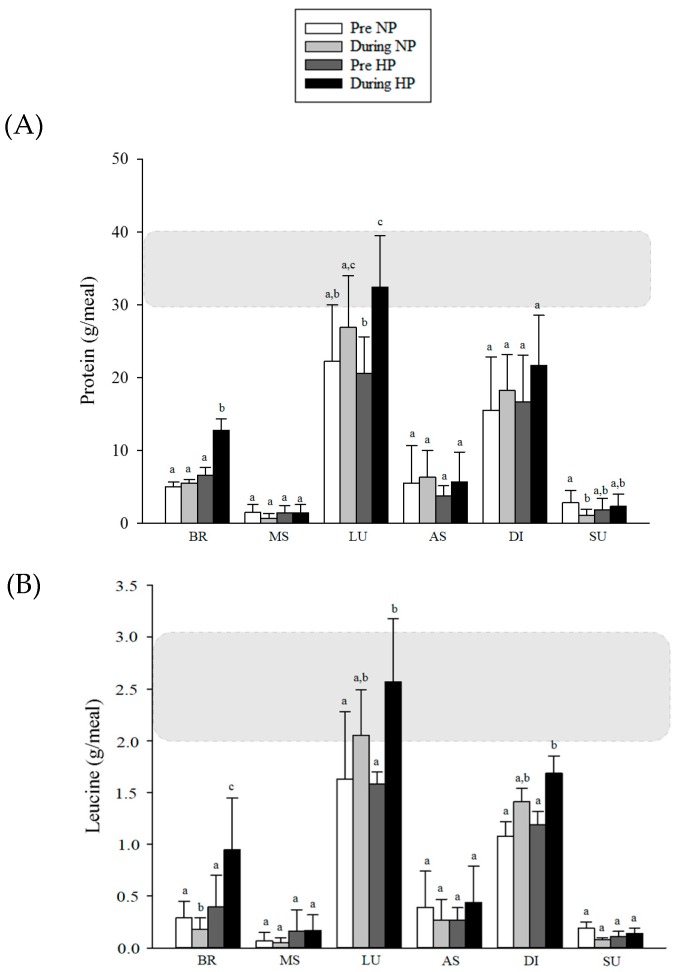
Distribution of protein (**A**) and leucine (**B**) according to meals of participants in the study. Notes: BR: breakfast; MS: morning snack; LU: lunch; AS: afternoon snack; DI: dinner; SU: supper. The darker rectangle in the graph represents the ideal amount that volunteers should achieve in consumption. Generalized Estimating Equation (GEE) with sequential Sidak post hoc. Different letters mean *p* < 0.05.

An increase in protein intake was observed at breakfast in the HP group in comparison to baseline (6.56 ± 1.08 vs. 12.72 ± 1.61 g, *p* < 0.001), and this value was also higher than the NP group during the study (12.72 ± 1.61 vs. 5.52 ± 0.50 g, *p* = 0.001). At lunch, the HP group showed higher protein intake compared to baseline (20.59 ± 5.01 vs. 32.41 ± 7.08 g, *p* < 0.001), and reached the recommended protein intake (~30 g per meal). However, the amount consumed by the HP group was not significantly different from the NP group (32.41 ± 7.08 vs. 26.89 ± 7.12 g, *p* = 0.122, respectively). At supper, the NP group decreased their protein intake during the study (2.85 ± 1.62 vs. 1.09 ± 0.84 g, *p* = 0.001), but no difference was found between groups (*p* = 0.085). For the other meals, no significant differences were observed between groups and moments. For leucine intake, a reduction in NP group (0.29 ± 0.16 vs. 0.18 ± 0.11 g, *p* = 0.024) and an increase in the HP (0.40 ± 0.30 vs. 0.95 ± 0.50 g, *p* = 0.001) at breakfast (time/group interaction; *p* < 0.001) was noted. At lunch and dinner, HP group increased the intake of leucine during the study (1.58 ± 0.12 vs. 2.57 ± 0.61 g, *p* < 0.001; 1.19 ± 0.13 vs. 1.69 ± 0.16 g, *p* = 0.17; respectively), but no difference was found between groups (*p* > 0.05). For the other meals, no significant differences were observed between groups and moments (Figure 4).

Additionally, no differences were found for protein intake before and after resistance training in both groups during the study. The protein intake before (~3 h) exercise was 7.02 ± 3.61 and 9.43 ± 5.1 g (*p* = 0.201) and after exercise (~3 h) was 21.7 ± 7.1 and 29.2 ± 12.2 g (*p* = 0.081) for NP and HP, respectively.

### 3.4. Strength

After intervention, HP group increased bench press and leg extension RM; however, no differences were found between groups and time x treatment (Table A1). No differences were found for Δ bench press RM (2.75 ± 3.01 vs. 1.09 ± 2.25 kg, *p* = 0.153) and Δ leg extension RM (6.75 ± 12.46 vs. 9.45 ± 7.03 kg, *p* = 0.533) for NP and HP group, respectively.

### 3.5. Body Composition

**Figure 5:**
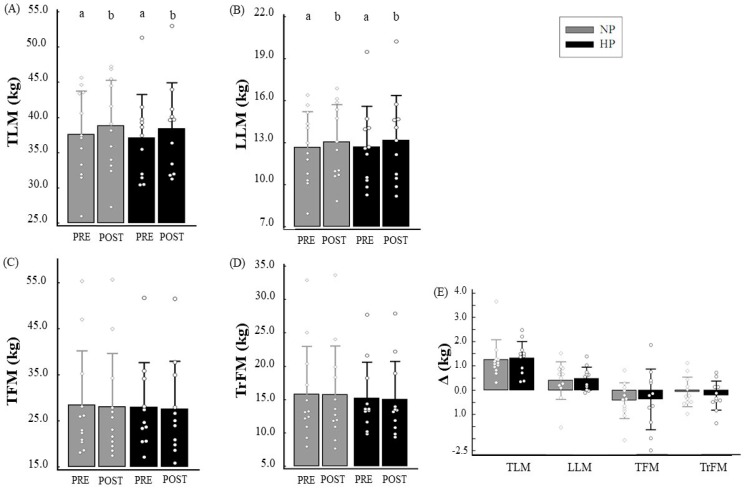
Total lean mass (TLM) (**A**), leg lean mass (LLM) (**B**), total fat mass (TFM) (**C**), and trunk fat mass (TrFM) (**D**) according to time and groups, and their respective deltas (**E**). Notes: Δ: delta values; Generalized Estimating Equation (GEE) with sequential Sidak post hoc was performed to evaluate the intake between the groups and during the study; independent *t*-test was performed to evaluate the difference between deltas. Different letters (a, b) mean *p* < 0.05.

Both the NP group (37.57 ± 6.17 vs. 38.83 ± 6.46 kg, *p* < 0.001; Δ = 1.26 ± 0.82 kg) and the HP group (37.10 ± 6.19 vs. 38.43 ± 6.49 kg, *p* < 0.001, Δ = 1.33 ± 0.68 kg) presented TLM gain after exercise protocol. Additionally, both groups increased LLM (NP: 12.66 ± 2.57 vs. 13.07 ± 2.67 kg, *p* = 0.003, Δ = 0.40 ± 0.77; and HP: 12.70 ± 2.89 vs. 13.19 ± 3.2 kg, *p* < 0.001, Δ = 0.48 ± 0.47), but no differences were found for Δ TLM and Δ LLM between groups. Regarding TFM and TrFM, no changes were observed in either groups (Figure 5).

In addition, after linear regression analysis, we found no association between Δ protein intake (g) and Δ total lean mass (kg) (*R*^2^ = 0.064; *β* = 0.253; *p* = 0.243) and Δ leg lean mass (kg) (*R*^2^ = 0.023; *β* = 0.153; *p* = 0.486) during the study.

## 4. Discussion

The main finding of the present study was that, contrary to our initial hypothesis, a higher protein intake (1.18 ± 0.3 g·kg^−1^·day^−1^) did not result in higher LBM gain when compared with an intake similar to that proposed by the RDA (0.87 ± 0.29 g·kg^−1^·day^−1^) after a 10-week resistance training protocol in postmenopausal women.

Recent studies have shown that protein intake recommended by the RDA seems to be insufficient to enhance the maximum MPS in elderly women and to maintain muscle mass during aging, therefore higher doses have been proposed [16,17,18,19,20,21]. This difference in protein dose recommendation is possibly due to the methodologies applied. Protein intake proposed by the RDA uses the nitrogen balance method, which has several limitations [38]. Currently, a new proposal for the recommended protein intake bases its methodology on the amino acid oxidation technique, which is an advanced, independent tracer-based method that circumvents many nitrogen balance limitations [18]. However, in our study, we did not find a greater LBM gain in the HP group, suggesting that the higher protein intake, which is close to new protein proposal/recommendations seems to have no additional benefits in long-term changes of LBM in older women.

The protein intake values compared in our study were obtained through the means of each group, and some individuals ingested higher or lower doses than the group average. However, this does not appear to be a limitation in our study because when the LBM gain of the participants that only ingested an amount equal to or less than 0.8 g·kg^−1^·day^−1^ was compared to those who ingested equal to or greater than 1.2 g·kg^−1^·day^−1^ the results remained the same (data not shown). In addition, we did not find an association between Δ protein intake (g) and Δ lean mass (kg), when evaluating all individuals together, which showed that the increase in protein intake had little to no effect on lean mass gain. Furthermore, the recommendations of protein intake for maximum muscle hypertrophy proposed by the American College of Sports Medicine position is 1.2–2.0 g·kg^−1^·day^−1^ [39]; therefore, we suggest that active postmenopausal women may need an even higher quantity of protein to promote higher LBM gains than offered in the present study. A study conducted by Tieland et al. evaluated the LBM gain in elderly men and women performing resistance training 2 times/week over 24 weeks [13]. The researchers observed a gain of ~1.3 kg of LBM in the group with consumption of ~1.3 g·kg^−1^·day^−1^ of protein (with protein supplementation), whereas in placebo (with consumption of ~0.9 g·kg^−1^·day^−1^ of protein), no LBM change was observed. In the present study, the postmenopausal women increased ~1.3 kg of LBM regardless of protein consumption. The differences between the studies for LBM gain can be explained by our younger sample age and the fact that the participants performed resistance exercise 3 times/week (6 sets of 8–12 repetitions), which contained exercises that recruited large muscle groups of upper and lower limbs. However, it is possible to suggest that if our volunteers were older, the intake of ~0.8 g·kg^−1^·day^−1^ of protein could promote lower LBM gain than higher protein intake, but new studies are needed to confirm it.

Besides total protein intake, the amount of protein intake per meal seems to be important for MPS and/or LBM gain, although this is not a consensus [35]. A balanced distribution of total protein intake with meals containing at least 30–40 g of high quality protein seems to be more effective in stimulating rates of MPS throughout the day in older individuals [40]. However, to the best of our knowledge, there are no chronic studies assessing the effect of protein distribution during the day on LBM gain in postmenopausal women, which is an important knowledge gap [41]. In our study, only the HP group reached this threshold at lunch (32.41 ± 7.08 g protein), whereas the NP group did not reach the recommendation at any meal. A research conducted by Farsijani et al. [42] demonstrated that over 2 years, people who consumed larger amounts of protein and more distributed throughout the day presented a greater amount of LBM. Another study found that more frequent consumption of meals containing 30–45 g of protein presents higher association with LLM and strength [43]. Therefore, only 1 meal reaching the recommended protein intake (as found in our study) may not be able to produce higher changes in LBM, and possibly, more meals reaching that threshold are needed, which increase the amount of protein intake per day.

Moreover, controlled experiments have elucidated that the amounts of protein containing 2–3 g of leucine can stimulate maximum MPS [44,45,46]. In our research, both the NP and HP groups reached the recommended amount of leucine per meal at lunch (2.05 ± 0.44 and 2.57 ± 0.61 g, respectively). In addition, the HP group increased the intake of leucine to ~2 g per day but this increase was distributed throughout the day, not just 1 meal. This did not appear to be sufficient to induce maximum MPS. McDonald et al. [16] demonstrated in a longitudinal study that older individuals (>65 years) ingesting 1.26 g protein·kg^−1^·day^−1^ and 7.10 g/day of leucine presented higher LBM maintenance over 6 years, whereas lower intake was associated with LBM loss. In our study, the HP group presented similar intake of both protein and leucine, but a higher increase in LBM was not observed when compared to lower protein intake group. Although an interventional study (present study) cannot be compared to a longitudinal study, it is possible to suggest that the length of time that ~1.2 g·kg^−1^·day^−1^ vs. ~0.8 g·kg^−1^·day^−1^ of protein is ingested may be important to promote greater gains and/or maintenance in LBM. Therefore, new interventional studies lasting more than 10 weeks that evaluate the effect of the RDA versus higher protein intake plus resistance exercise in LBM gain in postmenopausal women, are needed.

In our study, both groups increased LBM (~1.3 kg) similarly after the intervention, demonstrating that the resistance training was able to promote muscle hypertrophy in postmenopausal women after 10-weeks of training. This gain is in accordance with the literature, which shows that resistance training was effective in leading to muscular hypertrophy [47], including in postmenopausal women [8]. We found improvements in bench press RM and leg extension RM only in the HP group (independently of lean mass gain), but there were no differences in relation to the NP group when evaluating time-treatment interaction and Δ values. The same strength gains might have contributed similar LBM gains between groups.

We also found that higher protein intake did not result in changes of body fat. As we offered a normocaloric eating plan, the absence of fat loss was already expected. A normocaloric diet was prescribed because our main aim was to promote muscle hypertrophy and it is known that caloric restriction can attenuate this process [48]. In addition, there was no fat mass gain in the present study, which also seems important to promote maximum hypertrophy because the increase of trunk adiposity is correlated with lower LBM gain in postmenopausal women performing resistance training protocol [26].

The present study has limitations. An underreporting of dietary intake was observed in both groups, since the individuals related an energy intake similar to resting energy expenditure and no fat mass loss was observed. The postmenopausal women evaluated in the present study had high adiposity (~40% of body fat) and it was already shown that overweight and/or obese women underreport their energy intake [49]. This can be a limitation of the present study because the underreporting could be related to all macronutrients, including protein, which was the main dietary variable. However, it is possible to suggest that the protein intake was not affected by this underreporting because all dietary recalls were specifically focused on protein evaluation (daily and per meal), and the individuals reported a similar protein intake at baseline (~0.8 g/kg/day) than those found in other studies that evaluated postmenopausal women or older adults [13,50,51]. Furthermore, Lafay et al. [52] evaluated if energy intake underreporting occurs for specific food groups and found that certain food items rich in fat and carbohydrates such as butter, French fries, sugars, cakes, and biscuits were qualitatively underestimated. On the other hand, no difference was found for foods rich in protein when comparing underreported versus non-underreported individuals [52]; therefore, all these factors strengthen the proposition that protein intake was not underreported in our dietary data. However, even if an underreporting in protein intake had occurred, it would likely have occurred in both groups and we would continue to show that higher amounts of protein did not promote higher LBM gain. Another limitation was the large drop-out rate (~50%) in our sample; however, both groups presented similar losses during the study and no differences were observed between groups at baseline, which demonstrates the reliability of our data.

As for strengths of the present study, the protein intake before and after exercise was controlled between groups, since it seems that the timing of protein intake might be important for LBM gain [35]. In addition, indirect calorimetry was used to measure the resting energy expenditure of individuals for diet prescription. Additionally, our dietary intervention was based on increasing protein intake by a variety of protein sources, which represents a more realistic nutritional management in clinical practice.

## 5. Conclusions

In conclusion, the intake of higher amounts of protein (~1.2 g·kg^−1^·day^−1^) did not promote higher LBM gain when compared to the RDA (~0.8 g·kg^−1^·day^−1^) in postmenopausal women after resistance exercise protocol. Further studies with longer duration and a higher number of individuals are needed to evaluate the effect of these two protein intake recommendations on LBM gain in postmenopausal women

## Figures and Tables

**Table 1:** Baseline characteristics of the participants in the study.

Variables	NP	HP	*p*-Value
	*n* = 12	*n* = 11	
Age, years	63.0 ± 8.6	63.4 ± 7.6	0.895
*Anthropometrics*			
Weight, kg	69.0 ± 17.1	67.6 ± 15.6	0.840
Height, m	1.56 ± 0.08	1.55 ± 0.06	0.824
BMI, kg/m^2^	28.4 ± 6.0	28.1 ± 5.5	0.894
*Body Composition*			
Total lean mass, kg	37.6 ± 6.2	37.1 ± 6.2	0.857
Leg lean mass, kg	12.7 ± 2.6	12.7 ± 2.9	0.969
MMI, kg/m^2^	6.9 ± 0.9	6.9 ± 1.2	0.934
Total fat mass, kg *	24.5 (20.5–33.5)	27.7 (20.6–34.2)	0.786
Total fat mass, %	40.7 ± 7.0	40.9 ± 4.7	0.938
Trunk fat mass, kg *	13.2 (11.2–19.6)	13.6 (11.6–18.2)	0.740
*Strength*			
Bench press 1-RM (kg)	33.0 ± 4.9	32.2 ± 6.3	0.900
Leg extension 1-RM (kg)	71.1 ± 15.8	75.3 ± 14.8	0.518
*Biochemical parameters*			
Creatinine, mg/dL	0.78 ± 0.16	0.80 ± 0.18	0.768
Urea, mg/dL *	27.7 (24.4–35.8)	34.1 (30.3–36.0)	0.104
*Energy expenditure*			
REE, kcal	1582 ± 566	1394 ± 291	0.324
TEE, kcal	2127 ± 750	1881 ± 388	0.332

Notes: BMI: body mass index; MMI: muscle mass index, was calculated by lean mass (kg) divided by height squared; REE: rest energy expenditure; TEE: total energy expenditure; *: data with non-normal distribution. Independent *t*-test or Mann-Whitney; mean ± SD or median (interquartile range).

**Table 2:** Intake of calories, macronutrients, and BCAAs according to moments and groups.

Variables	NP (*n* = 12)		HP (*n* = 11)		*p*-Value		
	Pre	During	Pre	During	*Time*	*Intervention*	*Interaction*
Calories, kcal	1342 ± 423 ^a^	1439 ± 205 ^a^	1412 ± 382 ^a^	1502 ± 214 ^a^	0.267	0.494	0.968
Carbohydrate, g	161.6 ± 54.4 ^a^	168.7 ± 31.0 ^a^	182.3 ± 52.6 ^a^	179.9 ± 33.3 ^a^	0.829	0.237	0.668
Carbohydrate, %	48.4 ± 8.1 ^a,b^	46.9 ± 6.2 ^a,b^	51.5 ± 3.7 ^a^	47.7 ± 3.6 ^b^	0.024	0.296	0.326
Lipids, g	51.9 ± 16.7	56.4 ± 12.5 ^a^	53.9 ± 16.5 ^a^	53.3 ± 10.1 ^a^	0.642	0.895	0.535
Lipids, %	35.0 ± 6.3	35.4 ± 6.9 ^a^	34.1 ± 2.7 ^a^	32.0 ± 3.1 ^a^	0.400	0.219	0.205
Protein, g	51.5 ± 15.3 ^a^	58.7 ± 10.4 ^a^	53.2 ± 13.5 ^a^	77.4 ± 10.5 ^b^	<0.001	0.012	0.004
Protein, %	15.5 ± 2.9 ^a^	16.4 ± 2.2 ^a^	15.3 ± 2.7 ^a^	20.6 ± 2.6 ^b^	<0.001	0.009	<0.001
Isoleucine, g *	1.9 ± 0.6 ^a^	2.4 ± 0.5 ^b,c^	2.1 ± 0.5 ^a,c^	3.4 ± 0.5 ^d^	<0.001	0.008	0.011
Valine, g	3.4 ± 1.0 ^a^	3.4 ± 0.7 ^a^	3.6 ± 0.9 ^a^	4.9 ± 0.8 ^b^	0.002	0.009	0.008

Notes: BCAA: branched-chain amino acid. Generalized Estimating Equation (GEE) with sequential Sidak post hoc; all values are mean ± SD; *: data with non-normal distribution. Different letters (a, b) mean *p* < 0.05.

## Appendix A

**Table A1:** Strength tests values according to moments and groups.

Variables	NP (*n* = 12)		HP (*n* = 11)		*p*-Value		
	Pre	Post	Pre	Post	*Time*	*Treatment*	*Time × Treatment*
*Strength*							
Bench press RM (kg)	33.0 ± 4.9 ^a,b^	35.5 ±5.6 ^a,b^	32.2 ± 6.3 ^a^	34.3 ± 7.9 ^b^	0.002	0.644	0.215
Leg extension RM (kg)	71.1 ± 15.8 ^a,b^	77.8 ± 22.2 ^a,b^	75.3 ± 14.8 ^a^	84.7 ± 14.6 ^b^	<0.001	0.394	0.498

Different letters (a, b) mean *p* < 0.05.
